# Design of a Novel Multifunction Decision Support Display for Anesthesia Care: AlertWatch® OR

**DOI:** 10.1186/s12871-018-0478-8

**Published:** 2018-02-05

**Authors:** Kevin K. Tremper, Jenny J. Mace, Jan M. Gombert, Theodore T. Tremper, Justin F. Adams, James P. Bagian

**Affiliations:** 1 Department of Anesthesiology, University of Michigan Medicine, 1500 E. Medical Center Drive, Ann Arbor, MI 48109 USA; 2 AlertWatch Headquarters, 330 E. Liberty Street, Ann Arbor, MI 48104 USA

**Keywords:** Decision support, Integrated medical display, Anesthesia alerting system

## Abstract

**Background:**

This paper describes the design of a multifunction alerting display for intraoperative anesthetic care. The design was inspired by the multifunction primary flight display used in modern aviation.

**Results:**

The display retrieves live data from multiple sources; the physiologic monitors, the anesthesia information management system, the laboratory values and comorbidities from patient’s problem summary list, medical history or history & physical. This information is integrated into a display composed of readily identifiable icons of organ systems, which are color coded to signify normal range, marginal range, abnormal range (by green, yellow, red respectively) and orange outlines for comorbidities/risk factors. There are dozens of text alerts, which can be presented as black text (informational), red text (important information) and red scrolling text (highest importance information). The alerts are derived from current standards in the literature and some involve complex calculations being conducted in the background.

**Conclusions:**

The goal of such a system is to improve the quality and safety of anesthetic care by providing enhanced situational awareness in a fashion analogous to the “glass cockpit” and its primary flight display which has improved aviation safety.

**Electronic supplementary material:**

The online version of this article (10.1186/s12871-018-0478-8) contains supplementary material, which is available to authorized users.

## Background

**Fig. 1:**
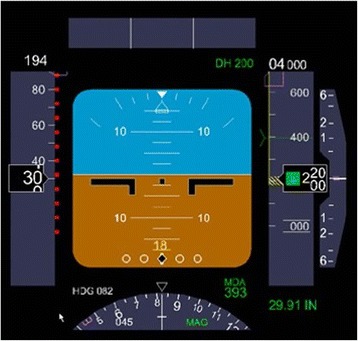
The Multifunction Monitor. In the 1980s aviation switched from multiple analog gauges to the “glass cockpit” with a multifunction monitor as the primary flight display. Above is the primary fight flight display of the Boeing 737 aircraft. It presents the horizon (blue sky/brown earth) to provide a visual assessment of yawl and pitch. There are also algorithms analyzing data which it presents when needed in the hierarchy of importance. [Primary Flight Display. (Accessed on August 9, 2016, at https://en.wikipedia.org/wiki/Primary_flight_display)]

The practice of anesthesia is often described in terms similar to that of being a pilot; the preop evaluation is the flight plan, the induction is the take-off, the performance of the procedure is cruise and the emergence is the landing. During each of these phases and during the “flight” the pilot/anesthesiologist follows the flight path of the procedure by relying on an array of instruments providing live data to assess the stability of the aircraft/patient. The aviation industry has led all industries in the area of safety and quality [1]. They have also led in the design of the cockpit from an increasing array of analog dials presenting data from an increasing number of sensors, to a more functional and intuitive display known as the multifunctional display or “glass cockpit [2].” This glass cockpit consolidates information from hundreds of sensors to three displays and has been associated with a significant improvement in aviation safety [1]. The three displays are the Navigation, the System’s Operations (displaying hydraulic pressures and other systems issues on the aircraft), and in front of the pilot and co-pilot, the multifunction display known as the Primary Flight Display. This integrated display provides an increased level of situational awareness by showing an integrated representation of the horizon, the ground, sky, which changes attitude showing pitch and roll of the aircraft, and a limited but important number of digital data such as airspeed and altitude. Figure 1 In the background multiple inputs of data are analyzed and presented when needed, in the order of importance. This system has enabled pilots to fly the plane and simultaneously address issues, which allows them to maintain “situational awareness” even during unplanned emergency situations. Medicine, and Anesthesiology in particular, has again followed the aviation industry with advanced monitoring standards, emergency protocols, team training, simulators and checklists; not only in the operating room but in all procedural areas [3–5].

With the broad implementation of the electronic medical record systems, which for anesthesiologists is known as the Anesthesia Information Management System (AIMS), anesthesiologists may now manage live digital data provided continuously and permanently stored. The proliferation of AIMS and other structured electronic medical record (EMR) systems, which include patient co-morbidities and outcomes in addition to intraoperative data, has resulted in a proliferation of observational studies finding associations between types of intraoperative care and postoperative outcomes [6–13]. The results of these studies may be applied in real-time during anesthesia care, but only if the information is made available to the provider at the time it is appropriate. That is, algorithms detecting labs, co-morbidities and physiologic data, which fall out of agreed upon standards can automatically trigger alerts in real time. As a result, live analysis can provide alerts to improve care in real time when there is the opportunity to improve the care of the patient, where reoccurring retrospective quality assessments may fall short. Examples including, but not limited to, glucose, blood pressure, ventilation and overall fluid management which can be assisted by live calculations and live alerts [6–13].

**Fig. 2:**
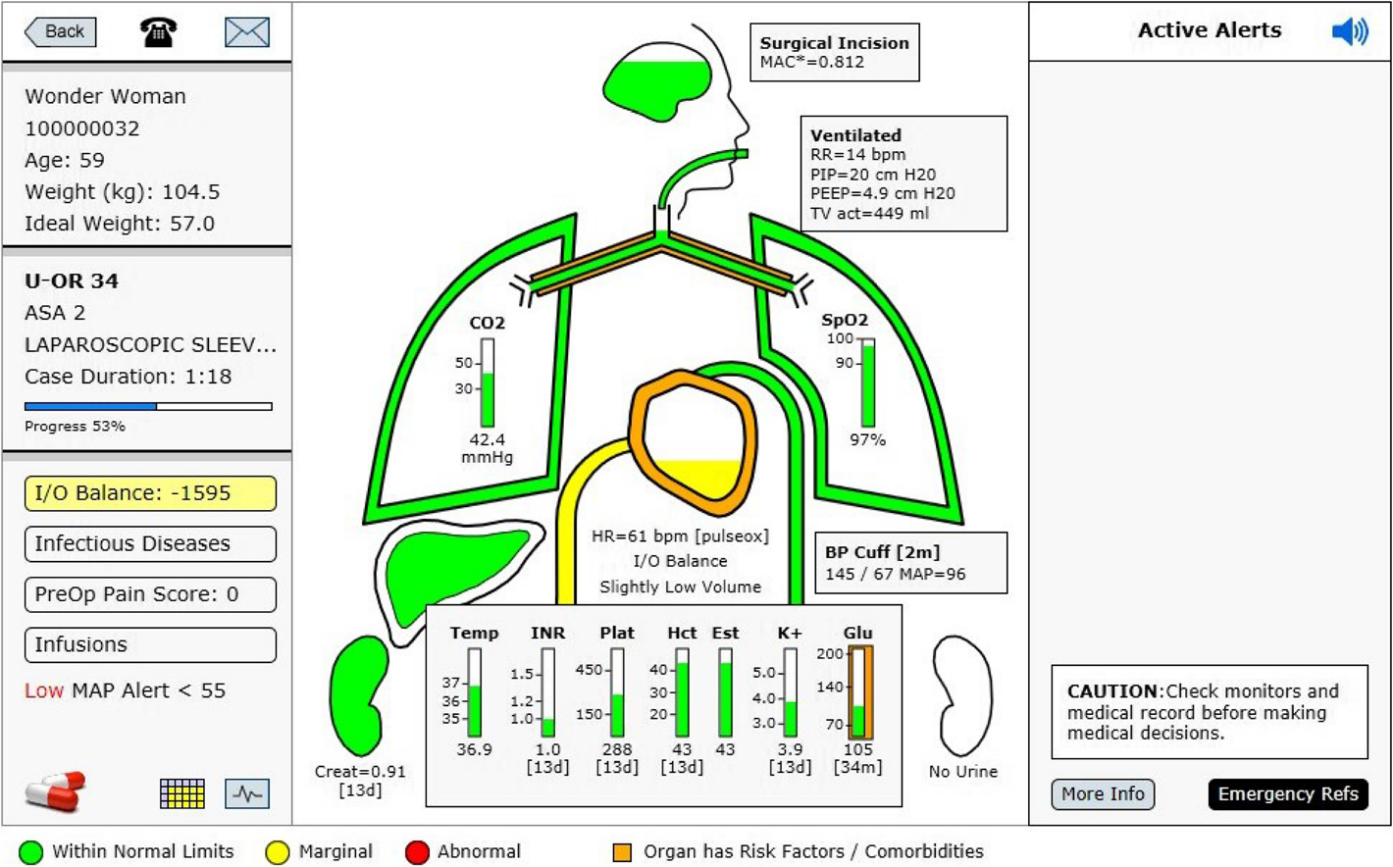
The AlertWatch® Display. The above AlertWatch® display is analogous to the primary flight display in aviation, Fig. 1. It receives information from physiologic monitors, AIMS, patients’ history and physical for co-morbidities and laboratory systems and displays this information in readily identifiable icons of human organs. It is color coded to signify: normal range (green), marginal (yellow), and abnormal range (red). Organs highlighted in orange have comorbidities. The left-side of the display provides patient case data, calculated intake and output (I&O) balance, presence of infectious disease, preoperative pain score and the presence of infusions. Text alerts are presented on right-side of the display in the following hierarchy: black text are informational, red text for important alerts and scrolling red text for those which need immediate attention, this figure has no text alerts, see Fig. 4a. There are 54 alerts originating from the 250 pieces of information continuously being extracted from the medical records. No data are stored in AlertWatch®, just retrieved from the EMR and presented. The central portion of the display is composed of icons of organ systems: brain, lungs, heart, liver kidneys. The color of the brain and level within the brain signifies the age-adjusted anesthetic level while the level within the heart signifies the fluid status with respect to I&O balance; green +/− 20% of estimated blood volume (EBV), yellow +/− 40% of EBV (high or low level) and red ≥40% EBV positive or negative (very high or very low level). This patient has an I&O of − 1595 so is in the yellow range and the heart is filled to the low yellow level. If there is an invasive arterial catheter or central venous catheter the fluid level and color within the heart will be guided by the normal ranges of SPV or CVP. The Green, Yellow and Red ranges for all variables are configurable. This patient has lung and cardiac disease signified by the orange outline. They also have diabetes noted by the orange outline of the glucose. All ranges/colors are presented in Additional File 1. For a more complete description go to the website go to the website: http://www.alertwatch.com

In an attempt to imitate what has been found successful in the aviation industry a multifunction display has been developed for operating room anesthesia [14–16]. AlertWatch® OR is a multifunction display, driven by live data extracted from physiologic monitors and the EMR, displayed in a readily identifiable icon view of organ systems, Fig. 2 [14, 15]. This display extracts, analyzes and presents over 250 pieces of information, providing a “live” organ system view with a beating heart, expanding/contracting aortic arch and ventilating lungs. The display is color coded to indicate normal range (green), marginal range (yellow), and abnormal range (red) for the data related to each organ system or lab value. Organs or systems with co-morbidities are outlined in orange. The display also has dozens of digital text alerts and two audible alerts (e.g. low or missing blood pressure). The text alerts which appear in the upper right alert section of the display come in three hierarchies: black text for basic information, red text for important information, and scrolling red text for information that should be addressed immediately. There are multiple calculations being conducted in the background for clinical purposes [17], AlertWatch® is a Food and Drug Administration (FDA) cleared decision support software device [17]. Because of the high incidence of colorblindness and as part of the FDA clearance process, the application has been tested using software that mimics colorblind individuals [18]. In addition, to ensure the orange co-morbidities are not confused with other color changes within organs and labs, the orange colors are highlighted around the organ or the lab, not filling the space thereby differentiating by the location. Most values and alerts are presented, not only by color changes, but digital changes and as stated above by scrolling text alerts and some audible alerts. The purpose of this current report is to present the design specifications and rational for display elements, algorithms, calculations and alerts in AlertWatch® OR version 2.60. As software development is an iterative process, elements of the AlertWatch system as described in this report are subject to change in future updates. A demo of the system can be viewed on the website: http://www.alertwatch.com/

## Implementation

The primary purpose of AlertWatch® is to extract data from multiple sources and display these inputs and other analyzed information in a readily interpretable manner so that clinicians can be made aware of the ever changing status of any patient in real-time. These live data extracts come from multiple sources within the hospital systems including: the admission & discharge system, physiologic data, the laboratory system, AIMS event documentation, as well as co-morbidities from the anesthesia history & physical or diagnosis codes from the patient’s problem list.

The physiologic data may be captured in a number of ways depending on institutional preference. Data retrieved from AIMS are updated less frequently than data captured directly from physiologic systems. The AIMS events and laboratory data are updated minute-by-minute and extracted from the AIMS and laboratory systems. The history & physical data, including coexisting diseases and airway exam, are extracted from the anesthesia history & physical or in some cases from standardized codes, such as international classification of diseases (ICD-10) or SNOMED. These comorbidities are categorized by organ system for display on the patient view, Fig. 2. Multiple types of data are extracted from the AIMS and utilized for calculations and display. These include fluid management information, anesthetic administration, ventilator data, etc. Each of these data extracts will be discussed in detail in sections below. AlertWatch® is a web-based decision support display, configurable to work with most AIMS and EMR systems.

## Results and design

### Census view

**Fig. 3:**
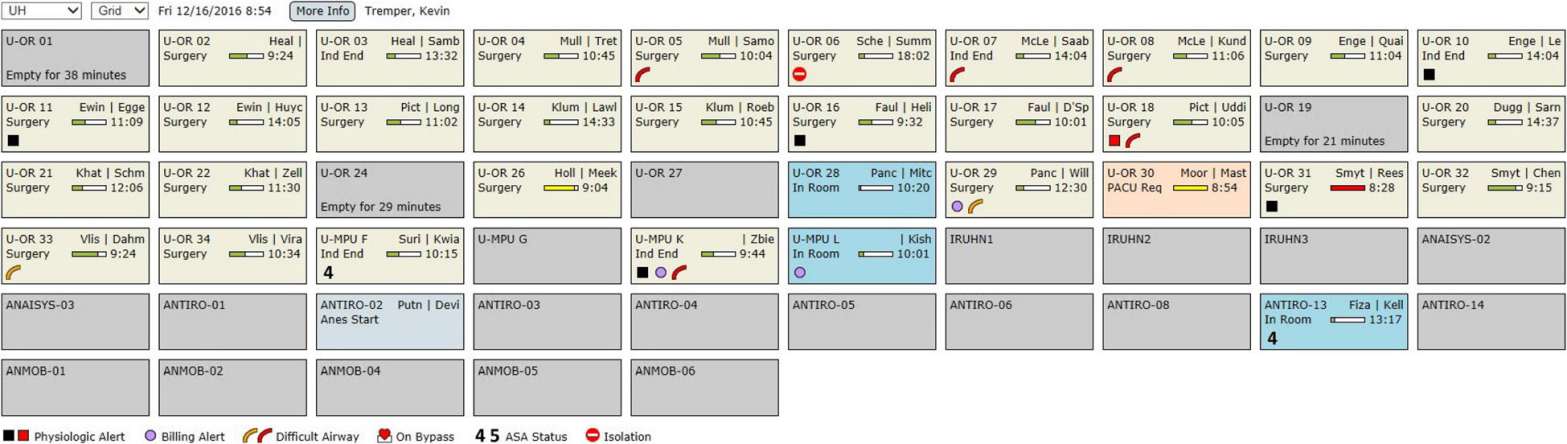
AlertWatch® Surveillance View. Above is the Surveillance/OR Census View of AlertWatch®; each square is an individual operating room. The anesthesia providers are in the upper left corner and the progress of the case is indicated by the colored scroll bar: green if on time, yellow if delayed, and red if delayed more than an hour. The background color of the square designates whether the patient is in the room/anesthesia start (blue), induction complete, surgery underway (tan), PACU called for (peach), or OR empty (gray). When the patient leaves the OR the room is gray and the number of minutes the OR has been empty is displayed. At the base of the OR rectangle icons indicate if there are text alerts (black or red), missing documentation, airway history, on cardiopulmonary bypass, ASA 4 or 5 and Isolation Precautions

AlertWatch® opens to a view of the OR census. This is a surveillance view that displays all the operating rooms being monitored, Fig. 3. Each rectangle represents an operating room (OR). If there are multiple sets of operating rooms they can be viewed by clicking on the upper left of the panel noting a drop-down list for all OR locations, Fig. 3. If a provider is logged into any cases at that time their census will only display their cases.

Each operating room rectangle provides information regarding OR number/location, the anesthesia providers in that OR and a track bar noting the case duration, Fig. 3. The track bar moves from left to right as the case proceeds and changes color depending on whether the case is on time (green) delayed (yellow) or very delayed (red), Fig. 3. The background color of the rectangle notes the status of the case: gray (no cases in the room), blue (anesthesia start), tan (induction end and surgery underway) and peach (post anesthesia care unit [PACU] called for). When the patient leaves the OR the number of minutes the OR has been empty is displayed, Fig. 3.

A series of icons are displayed in the lowest segment of the OR rectangle, Fig. 3. If there is an informational alert, a black square is displayed if there is a more urgent alert the square is red. A purple dot indicates that there is missing documentation. An orange elbow (representing the airway) represents risk factors for a difficult airway and a red elbow represents history of a difficult airway. The heart icon signifies the patient is on cardiopulmonary bypass. A number 4 or 5 represents the patient’s American Society of Anesthesiologists (ASA) status. Finally, the red circle icon with a white line through it represents isolation precautions. At the top left of center of the display there is a “More Info” button that links to a full user’s manual.

### Patient view

If you click on any OR icon an individual patient view from that OR is displayed. It has three distinct sections, the left side of the screen shows patient information, the right side of the screen shows active alerts, in the center is the live icon view of the patient’s organ system display “the primary flight display”, Fig. 2. Data are from real patients but all names and registration numbers are fictitious so the patients cannot be identified from these data. Additionally, no date of surgery is supplied.

The left section is the patient information, showing the patient’s name and registration number, age, weight and the calculated ideal body weight (calculated based on height and gender) [19]. In the upper left above the patient’s name is an icon of a telephone. It opens a window showing the anesthesia providers in the room with their pager numbers/phone numbers and the telephone number of the OR/Anesthesia machine, Fig. 2. To the right of the telephone is an envelope icon for sending messages to the AlertWatch® team for questions or suggestions.

In the section below the patient’s information is the OR number, the ASA status and the surgical procedure. Below the procedure is the case duration in hours and below that is a horizontal scroll bar designating the duration of the case relative to the schedule duration. Below the duration line is % of the case completed relative to the scheduled case time, Figs. 2 and 3.

Continuing down the far left side of the display is the I &O Balance, Infectious Disease, Preoperative Pain Score and Infusions, Fig. 2.

**Fig. 4:**
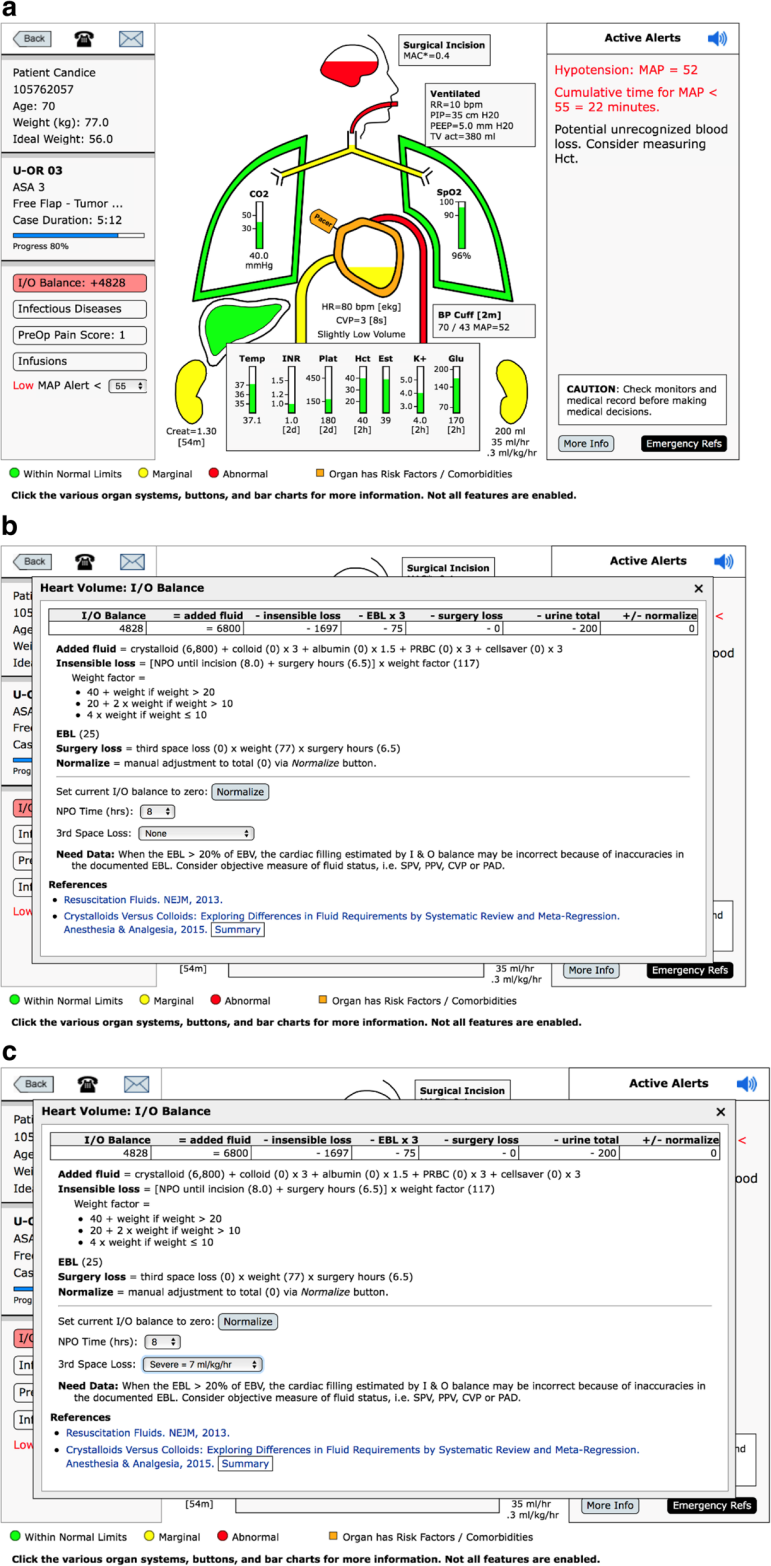
Patient View: Intake & Output (I&O). **a** The patient above has the I and O balance background of red because the calculated balance is ≥ 40% of the EBV positive, + 4828. When the I&O Balance button is opened, the window shows the itemization of the calculated balance, (**b**). This incorporates inputs of fluid minus outputs of fluid (losses). Losses are made up of insensible losses, estimated blood loss, urine output, and potential third-space surgical losses. The fluid balance is based on crystalloid, therefore, blood lost and blood transfused is weighted by three and albumin by 1.5. The insensible loss is calculated using the 4–2-1 rule and assumes 8 h of NPO. If that assumption is incorrect because the NPO time is different or the patient has had intravenous fluids, then that time can be corrected by opening the NPO scroll bar and picking the accurate NPO hours. If the patient is at a neutral fluid balance (in the opinion of the provider), the normalized button can be hit and the I&O calculation will be reset to zero. Note: the amount of fluid “normalized away”, either added or subtracted, is displayed on the far right end of the I&O calculation line. **c** The surgical “third-space loss” can range from 0 to 7 ml/kg/h depending on which level the user selects. Because most cases have relatively little third-space loss due to exposed surgical wounds and trauma, the default is set to zero. When the I&O window is open there is a user selection drop down menu for various levels of third-space loss, in this case severe = 7 ml/kg/h

If the calculated I&O balance is within 20% of the calculated estimated blood volume (EBV), the background color of that button is green [20]. If the calculated fluid balance is between +/− 20% to +/− 40% of EBV the background is yellow. If the calculated l&O balance is greater than 40% positive or negative relative to the EBV the background is red, Fig. 4a (also see Fig. 7a–c).

When you click the I&O button the detailed calculation is presented, Fig. 4b. The fluid losses (outputs) are: insensible loss, blood loss, third space surgical loss and urine output. The inputs are: crystalloids, colloids, blood and blood products. All ins and outs are based on crystalloid equivalents. Fluid administration with albumin, blood administration and loss are calculated in the following ratios relative to crystalloid, 1.5:1 and 3:1 respectively [21]. The insensible losses are estimated using the 4–2-1 rule. The 4–2-1 rule refers to the estimation of water losses due to respiration. The most common way of estimating this loss is to assume 4 ml/kg/h for the first 10 k, 2 ml/kg/h for the next 10 k and 1 ml/kg/h for the remaining kilos of body weight. The hourly water loss is multiplied by the NPO time. (22) The insensible loss defaults to 8 h of nothing by mouth (NPO). The actual hours of NPO can be selected from a drop down list, Fig. 4b. The surgical third space loss defaults to zero, but a drop-down list allows for a selection of three levels of loss; low (3 ml/kg/h), medium (5 ml/kg/h) and high (7 ml/kg/h), Fig. 4c [22].

The calculation assumes no intravenous fluid has been given prior to the start of the procedure and therefore maybe inaccurate, i.e. too negative. For that reason, (or any reason the clinical provider thinks the current I&O balance is zero) there is a “normalize button” which will “zero out” all the I&O calculations. If the normalize button has been activated the amount of fluids either positive or negative that has been “normalized away” is displayed on the far right of the I&O balance at the top. All windows within AlertWatch® have the links to the pertinent literature references at the bottom of the window. If there are no invasive measures of intravascular volume resuscitation being employed (e.g. SPV, CVP) then the heart icon will use the calculated I&O balance to determine the fluid level inside the heart icon, see next section, Fig. 2.

Below the I&O button is the Infectious Disease button. If the patient has an infectious disease or contact precaution that button will be highlighted in orange, and a red circle icon will be displayed on the census OR square. Selecting the Infectious Disease button displays the specific disease.

The next button is the PreOp Pain Score. If the score is ≥ 4, the button will be yellow; and if the patient has buprenorphine on their home medications list, the button will be orange.

The lowest button in this section identifies Infusions. If the patient is receiving an anesthetic infusion the button is colored green, if the patient is receiving a vasopressor or insulin infusion the button is yellow, and if the patient is receiving an epinephrine or high dose vasopressor infusions the button is red. Hitting this button will show the infusions.

At the bottom mean arterial pressure (MAP) low alert value is displayed. This can be changed by the provider on a case-by-case basis.

### Body icon view

The central portion of the display shows icons of the major organ systems, labs and the endotracheal tube. If an organ system or lab is colored / highlighted in orange that signifies a comorbidity associated with that organ system or lab, Fig. 2.

At the very bottom of the display the keys for green, yellow, and red; if that key is selected all the ranges will be displayed. These ranges are configurable on installation at an institutional level not for individual clinical providers, Additional File 1.

### Brain

The outline at the top of the figure is the brain icon. If the brain is highlighted in orange it designates there is history of a stroke or risk factors for stroke. Clicking the brain will open up a window showing the detail. Also in the window is a link to the reference used to determine the displayed minimum alveolar concentration (MAC*) value, discussed below. There are also links to emergency protocols.

**Fig. 5:**
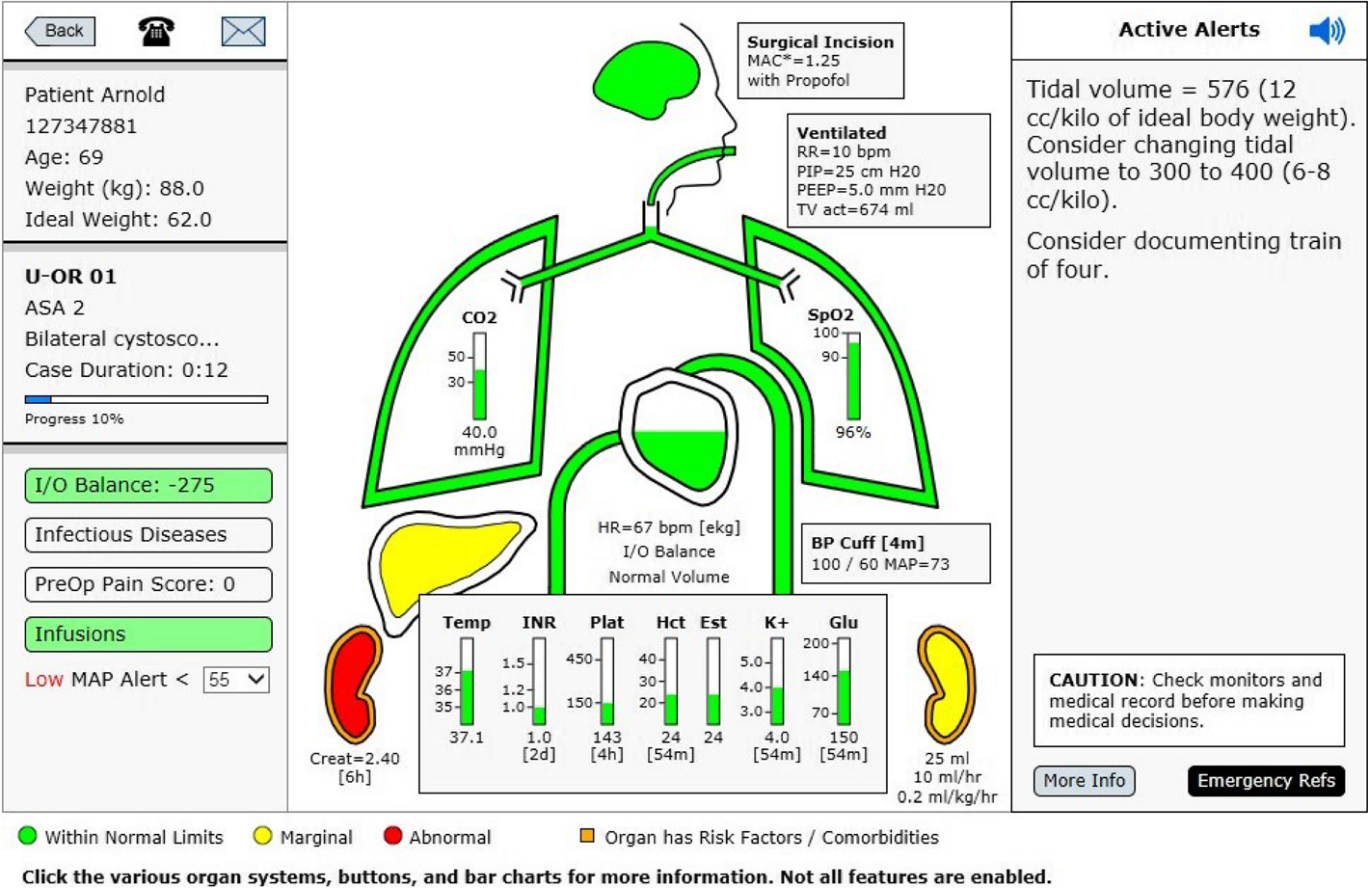
Brain Minimum Alveolar Concentration (MAC*) Level. The “fluid level” in the brain represents the level of anesthetic. When the calculated, age-adjusted MAC* level is ≥1.0 the brain color is green and full, Fig. 5. The level will lower until the MAC* level reaches 0.7, at which point the brain color will turn yellow, and as the level moves lower the brain will turn red when the MAC* is < 0.4, as in Fig. 4a. The MAC* is not the traditional MAC* associated with purely inhalation agents. It is the MAC* that has been associated with the incidence of postoperative recall and includes not only the additive value of inhaled anesthetic agents, but also intravenous agents such as propofol and dexmedetomidine [23, 24]

The “color level” within the brain is the calculated MAC* value, if the MAC* is equal to or greater than 1.0 the brain will be green and full, Fig. 5. As the MAC* decreases, the green level lowers until the MAC* is < 0.6. At this point the level turns yellow and when MAC* reduces to < 0.4 the level turns red, Fig. 4a. MAC* is a calculated anesthetic level associated with postoperative recall, i.e. MAC* represents a risk for recall not the traditional MAC calculation for inhaled anesthetics [23]. This cumulative MAC value for inhaled agents is age adjusted [23]. The equation for MAC* was used in a postoperative recall study conducted in three centers in over 22,000 patients [24]. Mashour, et al. determined the calculated MAC* value which was associated with an increasing incidence of patient recall. This calculated MAC* value incorporates both inhaled agents and intravenous agents. The inhaled anesthetic agents expired data are retrieved from the infrared analyzer on the anesthesia machine. The intravenous agents are received from the AIMS documentation of propofol and /or dexmedetomidine infusions. In addition, when an infusion anesthetic (propofol) is used in the MAC calculation it is stated in the MAC* box to the right of the brain, Fig. 5. There could be an error in over calculating the MAC if the infusion anesthetic were documented but discontinued, (pump problem, intravenous (IV) disconnect, etc.). Because this situation would be most severe if a patient was relaxed with a non-depolarizing muscle relaxant and had no inhaled agent, there is an alert on the display: “Pure TIVA documented. Neuromuscular blockade given. Consider bispectral index (BIS) or nitrous oxide”, Additional File 2, Category:Drugs. In addition, the large prospective trial referred to above excluded all patients under pure TIVA [24].

### Endotracheal tube (ETT)

**Fig. 6:**
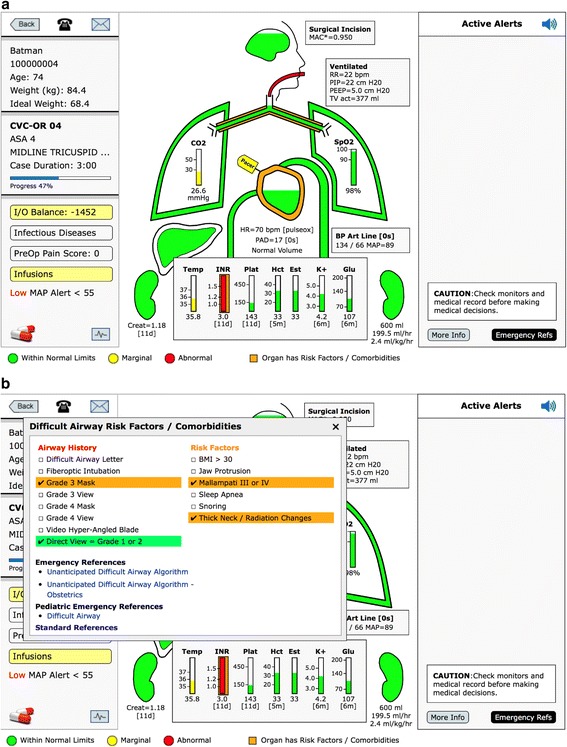
Endotracheal Tube (ET). **a** If there is a history of difficult mask ventilation (Grade 3 or 4) or intubation (Grade view 3 or 4) the ET tube will be red, as noted above. If there are risk factors for mask ventilation or intubation the ET tube will be orange. If there is a history of easy mask ventilation and intubation the ET tube will be green as noted in Fig. 5. **b** To see the specific findings, open the ET tube window. At the bottom of this window is a link to the algorithm for unanticipated difficult airway in adults, obstetrics, and pediatrics

The endotracheal tube (ETT) icon below the brain will appear clear in color if there is no airway assessment and no history of intubation, green if there is a history of easy mask (grade one or two) and easy intubation (grade one or two view) [25]. If there are risk factors for potential difficulty with airway management, including mask or difficult intubation, the endotracheal tube is orange. If there is a history of a difficult airway: (grade 3 or 4 mask or grade 3 or 4 view, use of an intubating stylet, use of video laryngoscopy or awake intubation) the ETT is red, Fig. 6a [26]. If you open the window of the ETT, the specific airway history and risk factors are displayed, Fig. 6b. There are also emergency references for unanticipated difficult airway in adults and pediatric patients along with literature references [27, 28].

### Ventilator data

If the patient is being ventilated with positive pressure ventilation, a box will appear to the right of the ETT showing the ventilation data: respiratory rate, peak inspiratory pressure (PIP), positive end expiratory pressure (PEEP), and tidal volume (TV), Fig. 5.

### Lungs

Below the ETT is the trachea and right and left main bronchia, Fig. 5. The lungs inflate and deflate with the respiratory rate. The color within the bronchus signifies PIP. If PIP is ≥35 cm H2O, it is yellow and ≥40 mmHg, it is red. The outline of the Lungs represents the PEEP, green with PEEP 0 to 10 (in adults there is a text alert if PEEP is < 4 cm H2O), yellow with PEEP ≥10 and < 15, and red when PEEP is ≥15 cm H2O. In the right lung is the saturation value (SpO2), and in the left lung is the end-tidal CO2 (ETCO2) value, Fig. 2.

If the trachea/bronchi are orange, the patient has a history of pulmonary disease. Clicking the trachea/bronchi highlights the pulmonary risk factors and pulmonary diseases e.g. asthma, chronic obstructive pulmonary disease (COPD), smoking history. It also has emergency protocols for bronchospasm and hypoxia as well as emergency pulmonary protocols for adults and children. If the TV is ≥10 ml/kg ideal body weight an alert will be displayed on the right with the recommended TV based on recent literature, Fig. 5 [8].

### Heart

**Fig. 7:**
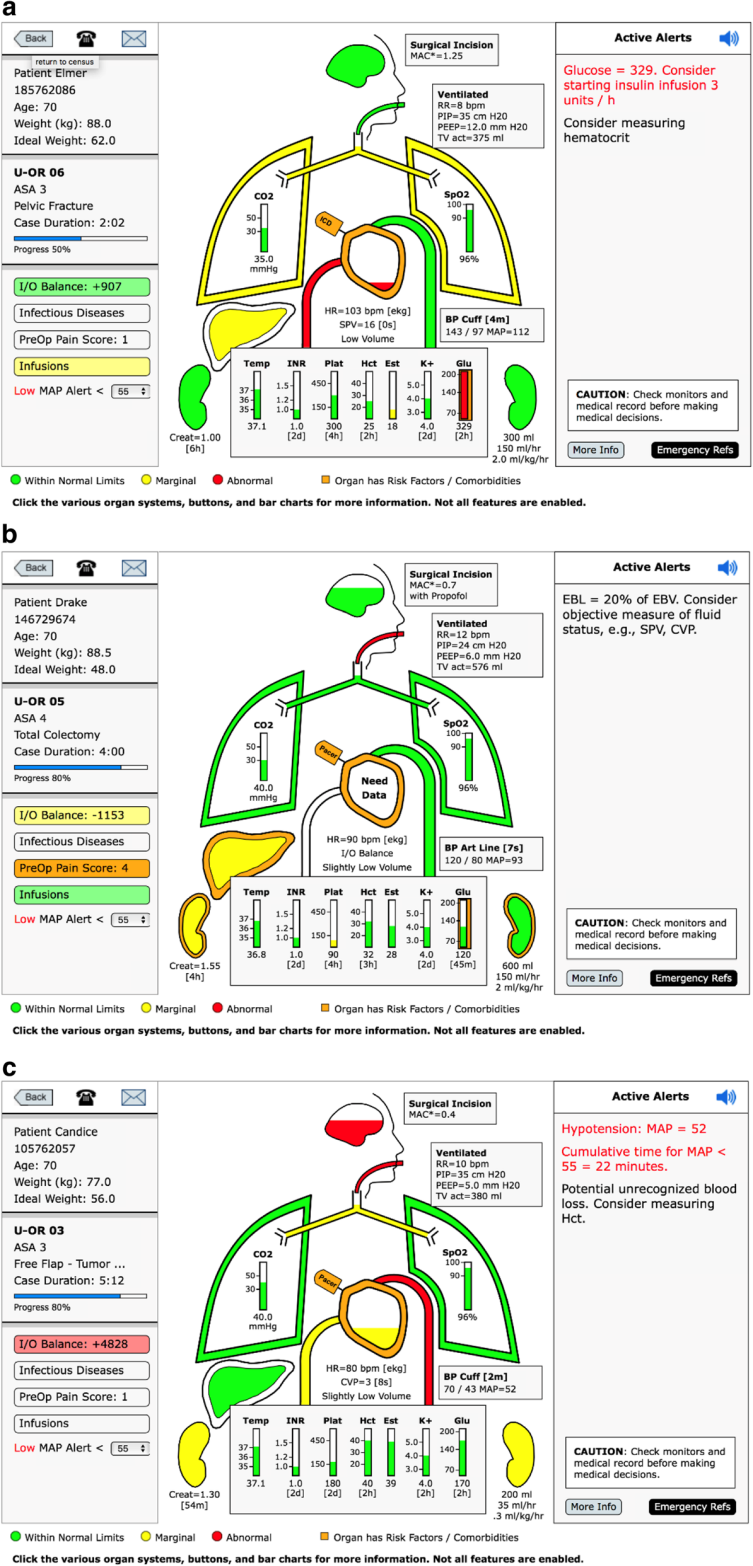
Heart. **a** The fluid level within the heart is represented as green, yellow, or red depending on the I&O balance unless there is an invasive measure of fluid status, such as, SPV or CVP. In Fig. 7a note the heart has an ICD (implantable cardiac defibrillator) represented by the small icon attached to the upper left side of the heart. **b** If there is no invasive assessment of cardiac filling and the estimated blood loss is ≥ 20% or the estimated blood volume the inside of the heart will not have a fluid level, but a text alert stating “Need Data.” A text alert in the upper right side of the display, stating “Consider Objective Measure of Fluid Status, e.g. SPV, CVP.”. **c** If the I&O balance is positive (≥ 60% of the EBV) but this coincides with an objective filling measures of SPV, PPV, CVP, or PAD all showing the heart has a very low filling volume and the most recent hematocrit is over 30 min old, a text alert will be triggered stating “Potential Unrecognized Blood Loss – Consider Measuring Hct”

**Fig. 8:**
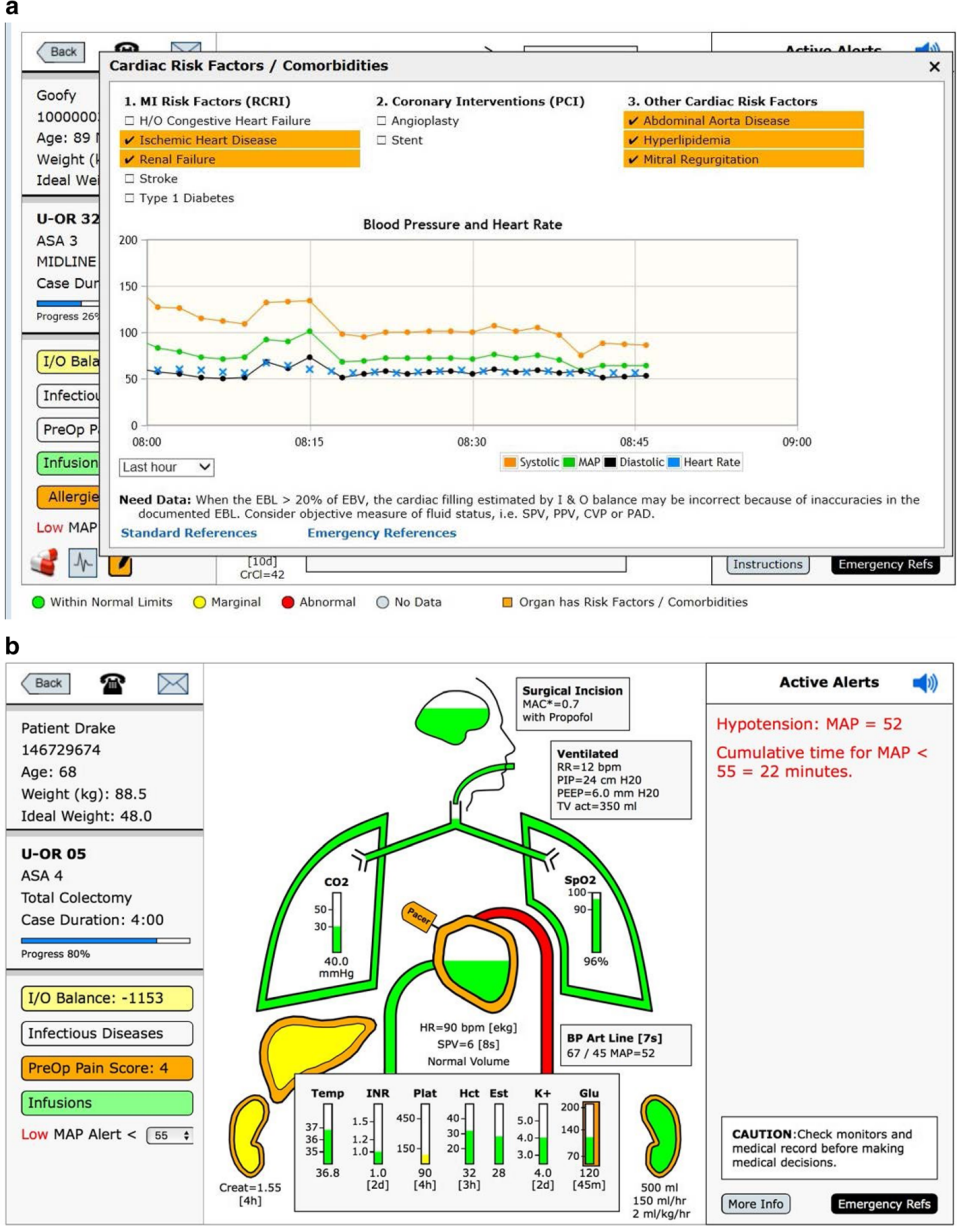
**a** Cardiac Risk Factors**.** Above the patient has cardiac co-morbidities and the heart is outlined in orange. When the heart is touched a cardiac co-morbidity window opens showing risk factors and co-morbidities. Under one are the revised cardiac risk index (RCRI) risk factors. To the left are other coronary interventions and to the left of that are other cardiac risk factors. The risk factors for this patient are highlighted in orange. **b**. Hypotension Color and Text Alert. Note above the aortic arch is red signifying the mean arterial pressure is < 55 mmHg. In the upper right side of the display a text alert signifies the blood pressure as well as the number of accumulated minutes the mean arterial pressure has been < 55 mmHg

The heart beats/contracts with the heart rate which is either driven from the electrocardiogram (EKG), if available, or the SpO2 pulse rate if the EKG is not available, Fig. 7a–c. If there is an orange outline on the heart it signifies comorbidity. Clicking on the heart icon displays the heart window which lists the Revised Cardiac Risk Indicators (RCRI) and highlights the factors present in this patient, Fig. 8a [29]. It also includes coronary interventions and other cardiac diseases. If the patient has a pacemaker or an implantable cardioverter defibrillator (ICD) there is a small icon attached to the heart, Fig. 7a and Fig. 8b. The window also includes links to adult and pediatric emergency cardiac protocol and references.

The fluid level within the heart is determined by the calculated fluid balance, I&O. The levels are described in the I&O section.

If there is an invasive method of assessing fluid status such as, systolic pressure variation (SPV), peak pulse pressure variation (PPV), central venous pressure (CVP), or pulmonary artery diastolic pressure (PAD), the fluid level within the heart will be guided by those values [30]. (See color limits in Additional File 1).

In cases where the estimated blood loss (EBL) is greater than 20% of the EBV and there is no objective measure of cardiac filling as mentioned above, then the area within the heart is white with the text “need data”. There is also a text alert in the upper right noting the high level of blood loss and suggesting the provider consider an invasive method of directly assessing fluid status, “Consider objective measure of fluid status, e.g. SPV, CVP,” or stroke volume variation (SVV) if available, Fig. 7b.

If the EBL is ≥40% of the EBV, the above alert is in red. In the situation where the I&O balance calculates a very positive, ≥ 60% of EBV but the objective filling measure (SPV, PPV, CVP or PAD) show a very empty heart and the last hematocrit (Hct) is over 30 min old an alert is activated: “Potential unrecognized blood loss. Consider measuring Hct”, Fig. 7c. There is a more in-depth discussion of all the alerts in the Text Alerts section. Systems may also choose to use hemoglobin (Hgb) instead of Hct as their site default.

On the left side of the heart is the icon of the inferior vena cava. Its color is always the same as the heart because the fluid level represents the filling of the heart.

### Blood pressure/aortic arch

The aortic arch coming out of the right side of the heart signifies the blood pressure (BP). It expands with the contraction of the heart at the heart rate. It is color-coded for BP, in the normal range is green when MAP ≥60 mmHg, yellow when MAP < 60 and red when MAP< 55 mmHg. At MAP < 55 mmHg there is also an audible alert of three tones of decreasing pitch, signifying a very low BP. This also triggers a red scrolling text alert in the upper right of the alerts section. When the cumulative time of MAP < 55 mmHg is ≥10 min there is a permanent red text alert at the top of the Alert Section showing with cumulative time the MAP has been < 55 mmHg for this case, Fig. 8b [7]. The actual BP is displayed along with age of the last BP measurement. When the BP has not been recorded for greater than 5 min there is alert and ≥10 min the BP measurement turns red.

### Liver

Under the right lung is the liver icon. If it is highlighted in orange, it means there is a history of hepatic disease, which is noted by opening the liver window. If the internal part of the liver is yellow, it means the liver function studies are out of the normal range, also shown in the window.

### Kidneys

On either side of the lab values are the kidneys. The kidney on the left side designates the chronic renal function, green if the creatinine is in the normal range (< 1.2 mg/dl), yellow if it’s marginal, creatinine ≥1.2 mg/dl but < 1.5 mg/dl, and red (abnormal) if the creatinine is ≥1.5 mg/dl. The kidney on the right of the display shows the current urine output in total ml/h. and ml/kg/h. If the ml/kg/h. < 0.5 mg/dl the kidney is yellow, Fig. 7c.

### Labs

Body temperature and laboratory values are at the bottom, Fig. 2. These labs all have green, yellow and red ranges which could be noted by selecting the icons at the far bottom of the screen, Additional File 1. The international normalized ratio (INR) and glucose columns maybe highlighted in orange if the patient has medications affecting the bleeding for INR, or if the patient has diabetes, respectively. The system will display a text alert of “No glucose recorded” if the patient is a diabetic.

**Fig. 9:**
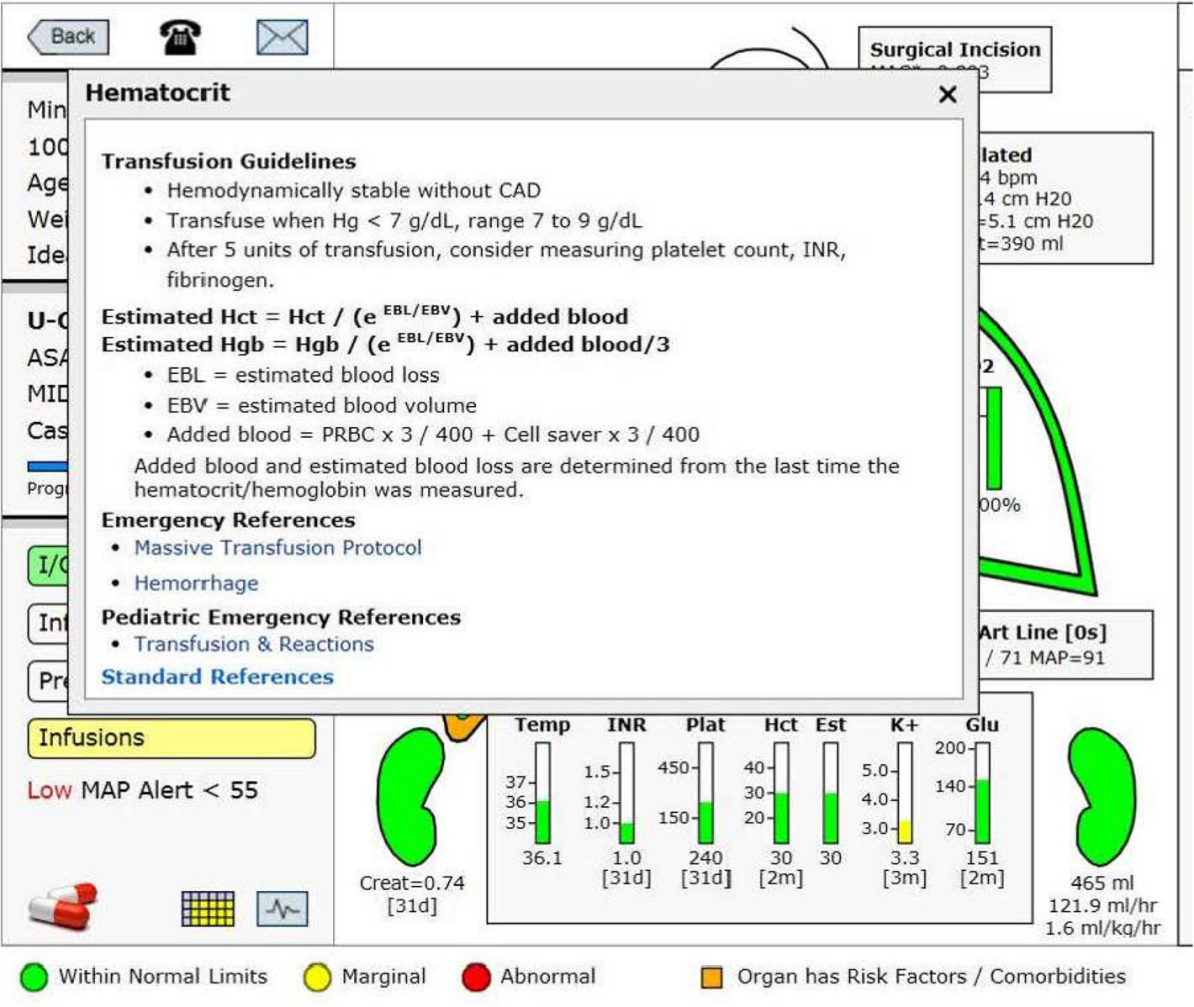
Estimated Hematocrit. This window opens when the estimated hematocrit or hematocrit columns are touched. It presents the equation used to determine an estimated hematocrit based on the last hematocrit drawn, the estimated blood loss, any blood given to the patient and the estimated blood volume, which is based on height, weight and gender. As soon as a new hematocrit is drawn and retrieved by the system the estimated hematocrit and hematocrit will be equal until there is a change in either blood loss or blood given to the patient

The Hct is displayed in the center with an estimated (Est) Hct to its right. The Est Hct is derived by equation using most recent Hct, EBV, EBL and blood transfused, see the equation in the window, Fig. 9 [20, 31].

### Active alerts

On the right side of the display is the active alert section. There are a total of 54 alerts, Additional File 2. These text alerts come in three levels of severity: black text are informational, red text are important and scrolling red text require an immediate attention. The vast majority of alerts are actionable items which when acted upon as in documenting the AIMS or by lab values retrieved, the alert disappears. There are two exceptions. When the MAP drops < 55 mmHg for more than 10 min the red alert stays in the upper right and presents the cumulative time the MAP has been below 55 mmHg, Fig. 8b [7]. The other alert which remains occur when bolus vasopressors are given. The rationale for having these alerts remain is to allow this information to be clearly noted/identified when there is a transition of care during a hand-over.

All other alerts are impacted by actions that are taken by the provider and will disappear when the appropriate action is taken. For example, if the last glucose measured in a patient record is over 200 mg/dl there will be an alert suggesting that a repeat glucose be measured. Once it has been measured if it is < 200 mg/dl the alert will disappear if it’s ≥ 200 mg/dl and the patient is not an outpatient and the case is longer than 1 h an alert will appear suggesting treatment with insulin [16]. Once there is documentation of insulin therapy that alert will disappear and a 60 min clock will start. At 60 min, if there is no repeat glucose lab noted an alert will appear recommending a repeat glucose measurement. This alert logic has recently been documented to improve glucose management [16]. Another example is if the TV is calculated to be ≥10 ml/kg ideal body weight there will be an alert displayed which suggests the appropriate TV to be within 6 to 8 ml/kg, Fig. 5 [8].

There are a series of alerts with respect to transfusion therapy suggesting when to measure labs/ coagulation studies, when to consider treatment with blood and blood products and calcium. These follow standard algorithms for transfusion therapy [32].

### Anesthesiology performance improvement and reporting exchange (ASPIRE) metrics

ASPIRE is a quality collaborative supported by Blue Cross Blue Shield of Michigan developed and coordinated by the University of Michigan. It is a collaborative of 30 hospital representatives of anesthesia departments in Michigan who jointly have determined a series of quality of care metrics [33]. AlertWatch® has coordinated its alerts with these ASPIRE quality metrics.

### Emergency references

Finally, in the lower right-hand corner of the screen is an emergency reference tab. This links to the adult emergency reference algorithms for the most common emergent events in the operating room [27].

### Configurable alerts and limits

The alerts and the limits on the alerts are all configurable upon installation at any institution. The FDA clearance process required the default alerts in the system have evidence in the literature and/or documented expert opinion in case the institution chooses to proceed with the default alerts and alert limits programed in AlertWatch OR.

## Discussion

The aviation industry is several decades ahead of medicine in taking advantage of large amounts of real-time data, to develop algorithms, protocols, and integrating them in a manner that increases situational awareness in order to improve safety and quality. This information is displayed in a readily identifiable format to enable the pilots to interpret and utilize these data while flying the aircraft: the primary flight display within the glass cockpit turns data into actionable information.

The explosion of medical data ranging from the EMR to the human genome, will provide the opportunity to develop more high resolution guidelines and protocols down to an individual patient level with their known comorbidities undergoing a specific procedure that can change on a minute to minute basis based on additional live data being received. To take advantage of this opportunity, there must be a way the care giver at the bedside can navigate these personalized recommendations and be alerted to these guidelines at the time, place and patient to which they should be applied. The increasing presence of monitors in critical care units has found that increasing the number of audible alerts to an array of analog waveforms with only simplistic high/low threshold alarms has frequently resulted in alarm fatigue rather than materially enhancing the care and safety of the patient [34].

One of the principles in developing AlertWatch OR was to provide an integrated display with a hierarchy of alerts presented in multiple, and some would say in a redundant manner; without producing alarm fatigue. This has been approached by presenting information in a hierarchy of color displays: red, yellow, green and orange; text alerts, black text, red text and scrolling red text; and some audible and/or paging alerts associated with the highest risk issues, e.g. severe hypotension, severe hypoglycemia.

Table 1 presents the number of alerts and categories of alerts for a seven-day period at the University of Michigan. As noted in this table, there were 5970 alerts in 1755 cases with an average case length of 126 min. Of those alerts, 1568 were for documentation and billing reminders, leaving 4402 alerts relating to all others aspects of the display. Drug dosing, antibiotic timing and train-of-four alerts accounted for 668 leaving 3734 more physiologic alerts. This one-week sample found approximately 2.5 text alerts per case, or at our institution 1.5 clinical text alerts per hour, Table 1. The most common of which was significant hypotension for greater than 10 min. Over this one-week period there were 314 red scrolling alerts associated with a page and/or a beep tone (2 glucose < 60 mg/dl, 9 hematocrit < 18%, and 303 mean blood pressure < 55 mmHg for 10 min) in approximately once every five cases.

**Table 1:** Text Alerts During One Week of Cases at University of Michigan ORs: 1755 cases

	Alerts	Alerts/Case	Alerts/Hour of Anesthesia
Total	5970	3.4	1.7
Documentation/Billing	1568	0.9	0.45
Clinical Alerts:	4402	2.5	1.25
Drugs/Antibiotics/Neuromuscular Blocker/ Train-of-Four	668	0.38	0.8
Glucose High/Low	118	0.07	
Cardiac	470	0.26	0.13
Blood Pressure	2466	1.4	0.7
Ventilation	589	0.3	0.15
Red Scrolling Text/Beeping Tone/Paging:	314	0.2	0.01
Glucose < 60 ml/dl	2	0.18	
HCT < 18%	9	0.01	
MAP < 55 mmHg (> 10 min)	303	0.17	0.08

Legend: Above are the total number of text alerts triggered by AlertWatch OR during seven days at the University of Michigan operating rooms. The total alerts at the top are 5970 alerts were triggered in 1755 cases. They are subdivided into documentation and billing alerts (the largest number, 1568) drug alerts, and physiologic alerts. At the bottom, the highest risk alerts, which are associated with scrolling red text and a beep tone or a page, are for low glucose, low hematocrit or mean blood pressure below 55 mmHg for more than 10 min. The most common of these serious alerts was for hypotension. AlertWatch® OR stores no clinical data. It only stores alerts for quality assurance purposes which are not part of the medical record

## Conclusion

AlertWatch® was developed over a five-year period at the University of Michigan. It was inspired by the aviation industry’s development of the primary flight display/multifunction display used in the glass cockpit display of modern aircraft. The aviation industry has led the way in safety. Although AlertWatch OR tries to emulate what has been found to be extremely useful in aviation, the physics of flying a plane are likely less variable than the physiology of managing a complex patient in the operating room. This is one reason why the AlertWatch OR icon display is more complex than the horizon display on a plane’s flight monitor, shown in Fig. 1. Clearly there is much more variability patient-to-patient and case-to-case and many of the “rules” are only as good as the most recent literature and guidelines on which those rules are based. The development of AlertWatch® is our attempt at emulating aviation’s processes to increase situational awareness with the goal of achieving their extremely high levels of safety.

## Availability and requirements

Project Name: AlertWatch OR.

Project Home Page: http://www.alertwatch.com

Operating System: Platform independent.

Programming Language: SQL/P-SQL, C#.NET with Java script front-end.

Other Requirements: Java enabled web browser.

License: No license required for viewing demo. FDA cleared software medical device requires licensing for installation.

Any restrictions to use by non-academics: No

## Additional files

Additional file 1: List of Color Limits. These color limits have received FDA Clearance, and they also can be changed at the institution level. (PDF 96 kb)

Additional file 2: List of Text Alerts. There are a total of 54 alerts. These text alerts come in three levels of severity: black text are informational, red text are important and scrolling red text require an immediate attention. (PDF 66 kb)

## Abbreviations

AIMS: Anesthesia Information Management System
ASA: American Society of Anesthesiologists
BIS: Bispectral index
BP: Blood pressure
COPD: Chronic obstructive pulmonary disease
CVP: Central venous pressure
EKG: Electrocardiogram
EMR: Electronic medical record
Est: Estimated
FDA: Food and Drug Administration
Hct: Hematocrit
Hgb: Hemoglobin
I&O: Intake and output
ICD: Implantable cardioverter defibrillator
ICD-10: International classification of diseases
INR: International normalized ratio
IV: Intravenous
MAC*: Minimum alveolar concentration
MAP: Mean arterial pressure
NPO: Nothing by mouth
OR: Operating room
PACU: Post Anesthesia Care Unit
PAD: Pulmonary artery diastolic pressure
PPV: Peak pulse pressure variation
SPV: Systolic pressure variation
SVV: Stroke volume variation
TIVA: Total intravenous anesthesia
