# Supplementary material for: Design of a Novel Multifunction Decision Support Display for Anesthesia Care: AlertWatch® OR
Source: BMC Anesthesiol. 2018 Feb 5;18:16. doi: 10.1186/s12871-018-0478-8 (PMC5799913; doi:10.1186/s12871-018-0478-8)
Supplement: Supplementary file 1 — List of Color Limits. These color limits have received FDA Clearance, and they also can be changed at the institution level. (PDF 96 kb) [file 12871_2018_478_MOESM1_ESM.pdf]

Additional File l. List of Color Limits

Area	Variable	Low Red	Low Yellow	Green	High Yellow	High Red
Brain	BIS	< 20	< 40	< 60	< 70	≥ 70
	MAC	< 0.5	< 0.7	≥ 0.7		
General	Glucose	< 60*	< 70	< 200	< 300	≥ 300
	Hematocrit	< 18*	< 24	≥ 24		
	Hemoglobin	< 6*	< 8	≥ 8		
	INR	< 0	< 0	< 1.3	< 1.5	≥ 1.5
	Platelet Count	< 50	< 100	≥ 100		
	Potassium	< 3	< 3.5	< 5	< 5.5	≥ 5.5
	Temperature	< 35	< 36	< 37.5	< 38	≥ 38
Heart	BP MAP	< 55*	< 60	< 130	< 150	≥ 150
	CVP	< 2	< 5	< 15	< 20	≥ 20
	I/O Balance (Fraction of EBV)	< -0.4	< -0.2	< 0.2	< 0.4	≥ 0.4
	PAP Dias	< 5	< 9	< 18	< 24	≥ 24
	PPV	> 19	> 15	> 7	> 3	≤ 3
	SPV	> 15	> 10	> 3.9	> 2	≤ 2
	SVV	> 20	> 15	> 10	> 5	≤ 5
Kidneys	Creatinine	< 0	< 0	< 1.2	< 1.5	≥ 1.5
	Urine	< 0	< 0.5	≥ 0.5		
Liver	Albumin	< 0	< 3	< 5.3	≥ 5.3	
	ALP	< 0	< 0	< 120	≥ 120	
	ALT / SGPT	< 0	< 0	< 52	≥ 52	
	Amylase	< 0	< 0	< 110	≥ 110	
	AST / SGOT	< 0	< 0	< 35	≥ 35	
	GGT	< 0	< 0	< 85	≥ 85	
	Lipase	< 0	< 0	< 60	≥ 60	
Lungs	CO2	< 20	< 30	< 50	< 60	≥ 60
	PEEP	< 0	< 0	< 11	< 15	≥ 15
	PIP	< 0	< 0	< 30	< 40	≥ 40
	SpO2	< 90	< 93	≥ 93		

Generally, a color (level) is assigned if the measured value is **less** than the entry in the table. High red is assigned when the measured value is **greater than or equal to** the entry for High Yellow. * Indicates flashing red square on census view, scrolling red alert on patient view and optional page to provider.
