# Supplementary material for: Design of a Novel Multifunction Decision Support Display for Anesthesia Care: AlertWatch® OR
Source: BMC Anesthesiol. 2018 Feb 5;18:16. doi: 10.1186/s12871-018-0478-8 (PMC5799913; doi:10.1186/s12871-018-0478-8)
Supplement: Supplementary file 2 — List of Text Alerts. There are a total of 54 alerts. These text alerts come in three levels of severity: black text are informational, red text are important and scrolling red text require an immediate attention. (PDF 66 kb) [file 12871_2018_478_MOESM2_ESM.pdf]

Additional File 2. List of Text Alerts

Category	Alert Description
Census	ASA 4
Census	ASA 5
Census	Patient on Bypass
Census	Isolation precaution
Drugs	Antibiotic last documented X minutes prior to surgical incision, where X ≥ 60 or 120, depending on the drug.
Drugs	Antibiotic last documented X hours ago, where X varies by drug.
Drugs	Antibiotic has not been documented prior to surgical incision.
Drugs	Pure TIVA documented. NMB given. Consider BIS or nitrous oxide.
Drugs	Consider documenting train of four.
Drugs	Reversal administered without TOF documentation.
Drugs	High cumulative vasopressor bolus dose, for vasopressors other than Epinephrine.
Drugs	High cumulative vasopressor bolus dose, for Epinephrine bolus.
Glucose	No glucose recorded (for patient with diabetes).
Glucose	Glucose = X, where X < 60.
Glucose	Glucose = X, where X ≥ 200. Consider starting insulin infusion 0.02 units / kg / hr, with a max of 1 unit / hr.
Glucose	Glucose = X, where X ≥ 300. Consider starting insulin infusion 0.04 units / kg / hr, with a max of 2 units / hr.
Glucose	Glucose = X, where X ≥ 350. Consider starting insulin infusion 0.06 units / kg / hr, with a max of 3 units / hr.
Glucose	Insulin infusion/bolus. Consider measuring glucose.
Heart	Hct = X, where X < 21. Consider transfusion.
Heart	Hct = X, where X < 18. Consider immediate transfusion
Heart	Consider measuring hematocrit, when estimated Hct < 21.
Heart	X% of EBV transfused. Consider checking coagulation profile. Treat with [Ca++, Cryoprecipitate, FFP, Platelets].
Heart	Ca++ given X minutes ago, where X ≥ 60. Consider measuring Ca++.
Heart	Cryoprecipitate given X minutes ago, where X ≥ 60. Consider measuring Fibrinogen.
Heart	FFP given X minutes ago, where X ≥ 60. Consider measuring INR.
Heart	Platelets given X minutes ago, where X ≥ 60. Consider measuring platelet count.
Heart	Ca++ = X mmol/L, where X < 1. Consider treating with 2 to 4 grams of calcium gluconate.
Heart	Fibrinogen = X mg/dL, where X < 100. Consider treating with cryoprecipitate.
Heart	INR = X, where X ≥ 1.5. Consider treating with FFP.
Heart	Platelet count = X, where X < 100k. Consider treating with platelets.
Heart	Consider measuring SPV or PPV.
Heart	SPV/PPV is X minutes old, where X ≥ 30.
Heart	EBL = X% of EBV, where X ≥ 20. Consider objective measure of fluid status, e.g., SPV, CVP.
Heart	EBL = X% of EBV, where X ≥ 40. Consider objective measure of fluid status, e.g., SPV, CVP.
Heart	Bradycardia, where heart rate below age defined limits.
Heart	Bradycardia, where heart rate below age-defined limits.
Heart	Tachycardia, where heart rate above age-defined limits.
Heart	Tachycardia, where heart rate above age-defined limits.
Heart	Potential unrecognized blood loss. Consider measuring Hct.
Hypotension	No BP Cuff measurement for more than 5 minutes.
Hypotension	No BP Cuff for 10 minutes or more.
Hypotension	No BP Cuff for 15 minutes or more for pediatric patients on a-line.
Hypotension	Potential hypotension, based on the slope of the last two measurements.
Hypotension	Hypotension: MAP = X, where X = 55 for adults or 2 x age + 25 for children.
Hypotension	Cumulative time for MAP < 55 more than 10 minutes (adults only).
Hypotension	Hypotension: MAP = X, where X = 60 for adults or 2 x age + 30 for children.
Lungs	PEEP = X, where X < 4.
Lungs	CO2 Rebreathing. Inspired CO2 = X mm Hg, where X ≥ 5.
Lungs	Tidal volume = X (Y cc/kilo of ideal body weight), where Y < 2 or y > 10. Consider changing tidal volume to 6-8 cc/kilo of ideal body weight.
Lungs	Tidal volume has been low/high for > 10 minutes.
Other	Potentially invalid data. Check primary source.
Other	You are in room X, but viewing room Y.

You can silence the alarm for the duration of the current case by clicking on the speaker icon. An **X** in a message is replaced with the current value. All text alerts have received FDA clearance.
*Scrolling Red Text
